# Real‐world use of inhaled treprostinil for lung disease‐pulmonary hypertension: A protocol for patient evaluation and prescribing

**DOI:** 10.1002/pul2.12126

**Published:** 2022-07-01

**Authors:** Shelsey W. Johnson, Lauren Finlay, Stephen C. Mathai, Ronald H. Goldstein, Bradley A. Maron

**Affiliations:** ^1^ Department of Pulmonary, Allergy, Sleep, and Critical Care Medicine, VA Boston Healthcare System Boston Massachusetts USA; ^2^ The Pulmonary Center, Division of Pulmonary, Allergy, Sleep and Critical Care Boston University School of Medicine Boston Massachusetts USA; ^3^ Department of Pharmacy VA Boston Healthcare System Boston Massachusetts USA; ^4^ Department of Pulmonary and Critical Care Medicine Johns Hopkins University, and Johns Hopkins School of Medicine Baltimore Maryland USA; ^5^ Division of Cardiovascular Medicine Brigham and Women's Hospital, and Harvard Medical School Boston Massachusetts USA

**Keywords:** Group 3 pulmonary hypertension, inhaled treprostinil, interstitial lung disease, pulmonary hypertension

## Abstract

Inhaled treprostinil was approved recently for interstitial lung disease‐pulmonary hypertension; however, efficacy in “real‐world” populations is not known. We designed a protocol and report our experience evaluating 10 patients referred for therapy. Misdiagnosis at presentation was common; ultimately, three patients (30%) were prescribed drug. This protocol offers an opportunity to standardize longitudinal assessment of inhaled treprostinil in clinical practice.

*To the Editor:*

In patients with interstitial lung disease (ILD) or chronic obstructive pulmonary disease (COPD), pulmonary hypertension (lung disease‐PH) is associated with diminished exercise capacity and quality of life compared to counterparts without PH. Among at‐risk patients, elevated mean pulmonary artery pressure (mPAP) is independently associated with lower 5‐year survival compared to normal mPAP (16.7% vs. 62.2%).^1^ Further, in the largest observational study to date involving 32,725 patients referred to right heart catheterization (RHC), ILD or COPD was identified in 36% of the cohort.^2^ Taken together, lung disease‐PH patients constitute a sizeable and highly vulnerable population and, thus, advancing pharmacotherapeutic options for affected patients is critical.

In April 2021, the US Food and Drug Administration (FDA) approved the use of inhaled treprostinil for the treatment of patients with ILD‐PH, representing the first authorized medical therapy for this patient population. This milestone achievement followed results from the INCREASE study,^3^ which demonstrated a + 31 m improvement in 6‐min walk distance (6MWD) from baseline at 16 weeks in patients with ILD‐PH treated with inhaled treprostinil compared to placebo (95% CI: 16.9–45.4 m; *p* < 0.001). The effect of inhaled treprostinil on 6MWD also drove a reduction in clinical worsening risk by 39% (95% CI: 0.40–0.92; *p* = 0.04) compared to placebo‐treated patients. Post‐hoc analyses suggest treatment benefit for disease progression^4^ and, unexpectedly, forced vital capacity.^5^ Collectively, these results have perpetuated enthusiasm for the use of inhaled treprostinil in clinical practice.^6^ However, frequent dosing, variable clinical response in individual patients, and high cost may be potential barriers to medication adherence outside of tightly regulated clinical trials. Indeed, guidance on the approach to evaluating efficacy of inhaled treprostinil in “real‐world” patients is needed but presently lacking.

To address this knowledge gap, we assembled a protocol for evaluating patients with lung disease‐PH referred for consideration of inhaled treprostinil therapy. This effort aims to establish guidance on clinical assessment for use of inhaled treprostinil and quantification of clinical benefit. Here, we outline this protocol and report our initial experience involving 10 patients evaluated for treatment following FDA approval of inhaled treprostinil.

## METHODS

As part of a quality care initiative and in line with reports suggesting multidisciplinary treatment approaches are optimal for PH care,^7^ we organized a working group at the VA Boston Healthcare System consisting of clinical PH experts in pulmonary and cardiovascular medicine, clinical pharmacy, and respiratory therapy to design a protocol for prescribing inhaled treprostinil to patients with lung disease‐PH. The appropriate inclusion and exclusion criteria, drug titration schedule, and outcomes used to document efficacy for our protocol were derived from the INCREASE study and other formative trials involving inhaled treprostinil.^3^, ^8^ Allowance for consideration of compassionate use prescribing in patients with COPD‐PH was based on two small studies demonstrating safety^9^ and efficacy,^10^ respectively, which have laid the groundwork for the ongoing Phase 3 PERFECT trial (NCT03794583 at clinicaltrials.gov) to prospectively evaluate inhaled treprostinil on outcome in COPD‐PH. Additionally, inclusion of patients with combined pulmonary fibrosis and emphysema in INCREASE (*n* = 82; 25.2%) substantiated consideration of compassionate use therapy in patients with a component of obstructive lung disease.^3^ The 10‐item emPHasis‐10 questionnaire, a validated health‐related quality of life (QOL) survey for pulmonary arterial hypertension (PAH) patients,^11^, ^12^ was selected based on its PH QOL assessment specificity (as compared to the chronic lung disease‐specific St. George's Respiratory Questionnaire used in INCREASE),^3^ as well as accessibility, and brevity.

Veterans referred by both intramural and extramural pulmonologists and cardiologists were then evaluated in our PH clinic using the approved protocol. Patient data were collected from the electronic medical record during each referral visit. Dichotomous variables are summarized as *N*(%) and nonnormally distributed continuous variables are reported as median (interquartile range). The protocol was approved by the Veterans Affairs Boston Healthcare System and case reporting of deidentified data was deemed exempt from detailed IRB review.

## RESULTS

### Protocol

Critical protocol components include invasive hemodynamic assessment with RHC, appraisal of patient‐capacity to learn and comply with proper inhaler technique, abstinence from inhaled tobacco or marijuana products, outpatient titration of therapy under prescribing clinician or specialty pharmacy supervision, and objective assessment of clinical response to therapy after 16 weeks (Table 1).

**Table 1:** Protocol for evaluation and initiation of inhaled treprostinil for real‐world lung disease‐pulmonary hypertension (PH) patients referred for consideration of therapy

Inclusion criteria	Exclusion criteria	Baseline physiologic and functional assessments	Dosing
***All** of the following must be selected for patient to be eligible:*	❑ Patient deemed unable to learn and/or comply with proper inhaler use as per pharmacy assessment ❑ Current use of any inhaled tobacco or marijuana products ❑ If alternate WHO clinical PH group is identified and/or there is concern for cardiogenic shock due to PH, treatment decisions should be made based on in multidisciplinary discussion regarding consideration of oral and/or intravenous therapies ❑ Prescription for alternate oral and/or parenteral PH therapy	❑ Full pulmonary function testing (PFT) within year of starting therapy ❑ Echocardiogram within 6 months of starting therapy ❑ Noncontrast CT chest within 6 months of starting therapy ❑ Physical exam by pulmonary provider with vital signs including heart rate, blood pressure, resting oxygen saturation ❑ WHO functional class assessment ❑ NT‐BNP ❑ 6‐min walk test (6MWT) with oxygen saturation assessment and Borg Dyspnea Score ❑ Emphasis‐10 survey to assess PH impact on quality of life	*All Tyvaso starts will be done **outpatient** with initiation and uptitration under the supervision of the prescribing physician, and, in some cases, specialty pharmacy. In particular, nurses will conduct home visits on the following schedule to assess treatment response: Weeks 1, 2, and 3 (in person), Week 4 (phone visit), Weeks 6 and 16 (in person)* Drug timing and frequency: 4 times daily, ~4 h apart during wake hours Starting dose: 3 breaths (6 mcg/inhalation) with target dose 9 breaths and maximum dose 12 breaths ▪ Anticipated dose titration: ▪ *Week 1*: 3 breaths, 4 times daily ▪ *Week 2*: 4 breaths, 4 times daily ▪ *Week 3*: 5 breaths, 4 times daily ▪ *Week 4*: 6 breaths, 4 times daily Dose titration will be supervised by specialty nursing with plan for increase of 1 additional breath every week (with room to increase by 2–3 additional breaths per week at the discretion of the prescribing physician). Specialty nurse will make prescribing physician aware of **all dose changes** via phone call. The prescribing physician may discontinue dose escalation at any time for adverse side effects.**
❑ Confirmed ILD by pulmonary clinician including outpatient pulmonary visit or inpatient pulmonary consult service ***OR*** for individual compassionate use consideration in patients with COPD pending multidisciplinary expert consensus discussion ❑ Confirmed diagnosis of pre‐capillary PH by RHC within past 6 months: mPAP ≥ 25 mmHg, PVR ≥ 3.0 WU*, PAWP ≤15 mmHg; CO (L/min) to be measured by thermodilution (rather than estimated by Fick) if possible ❑ Patient has been assessed for compliance with other background therapies (i.e., antifibrotics such as Pirfenidone, oxygen, CPAP); *to be assessed by pharmacists **before** proceeding with additional diagnostic testing* ❑ **Alternate** World Symposium clinical PH group **is not identified**			
		*Note: if Veteran is inpatient at time of evaluation for inhaled Treprostinil, the inpatient pulmonary fellow should be consulted who will assist in organizing PFT and 6MWT with results including WHO functional class assessment to be documented in pulmonary fellow consult note*	

**Follow‐up**			
ALL patients initiated on inhaled Tyvaso will be seen in person in clinic 16 weeks after starting therapy			
❑ Serial documentation of all parameters recommended in “baseline physiologic and functional assessments” should be repeated; peak 6 MWT and oxygen saturation evaluation is to be completed by respiratory therapy 10–60 min after the most recent dose			
❑ Documentation of adverse side effects** if any			
❑ Echocardiogram will be ordered at this follow‐up visit to document response after therapy initiation as per ESC‐ERS PAH guidelines			
*A positive response to inhaled treprostinil therapy will be defined by patients meeting any one or more of the following criteria*			
❑ ≥32‐m improvement in 6 MWD from baseline			
❑ WHO FC improvement to II wherever possible			
❑ Improvement in Borg dyspnea score on 6 MWT			
❑ ≥6‐point improvement on emphasis‐10 QOL survey			

* Note, data for use in ILD patients has demonstrated particular efficacy in patients with PVR ≥ 4 WU.

** Adverse side effects: cough (most common), headache, nausea, dizziness, flushing, throat irritation, diarrhea (*note trial data **did not** demonstrate worsening hypoxemia or increased need for supplemental oxygen*).

### Patient characteristics

In the 6 months following protocol approval, *N* = 10 patients with lung disease‐PH diagnosed by the referring physician were evaluated in our clinic for inhaled treprostinil therapy consideration. The study population included *N* = 9 males (90%) and the overall mean age was 75.5 (4.0) years. Four patients (40%) referred to our clinic had COPD, *N* = 2 (20%) had idiopathic pulmonary fibrosis (IPF), and *N* = 4 (40%) were identified to have overlapping obstructive and restrictive physiology with both COPD and non‐IPF ILD. At the time of referral, a total of *N* = 8 patients (80%) required supplemental oxygen at rest (2.5 [2.5] liters per minute) and *N* = 9 (90%) had undergone RHC within 6 months before referral (49 [195] days). One patient deferred recommended RHC. Half the patients (*N* = 5; 50%) who underwent RHC had cardiopulmonary hemodynamics consistent with isolated precapillary PH defined by mPAP ≥ 25 mmHg, pulmonary artery wedge pressure (PAWP) ≤ 15 mmHg, and pulmonary vascular resistance (PVR) ≥ 3 Wood units. Median mPAP for the cohort was 37.0 [14.0] mmHg with PAWP 10.0 [5.0] mmHg and PVR 3.9 [3.6] WU.

**Figure 1:**
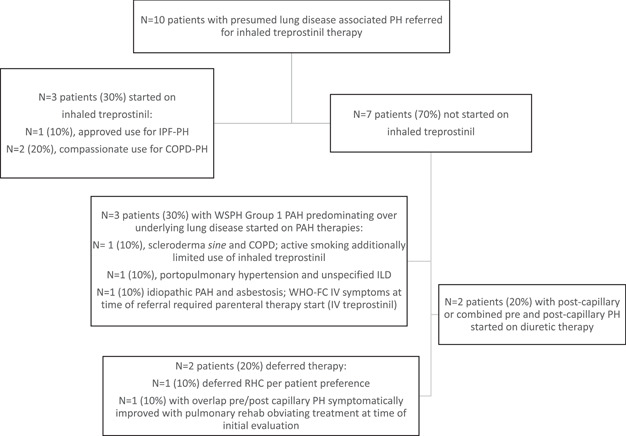
Consort diagram of treatment determinations for patients with presumed lung disease‐pulmonary hypertension (PH) referred for consideration of inhaled treprostinil therapy. COPD, chronic obstructive pulmonary disease; ILD, interstitial lung disease; IPF, idiopathic pulmonary fibrosis; PAH, pulmonary arterial hypertension; PH, pulmonary hypertension; RHC, right heart catheterization; WHO‐FC, World Health Organization‐Functional Class; WSPH, World Symposium on Pulmonary Hypertension.

Only *N* = 3 patients (30%) referred to our clinic were, in fact, initiated on therapy. These included two patients with COPD and one with IPF. Deferred prescribing of inhaled treprostinil was driven primarily by identification of alternate World Symposium on Pulmonary Hypertension (WSPH) Group classifications, outside of isolated lung disease‐PH, requiring different therapies. These determinations were adjudicated in a multidisciplinary format, following an approach outlined by other PH programs, including at other VA centers,^7^ and were supported by consideration to pivotal decision points abstracted from the WSPH chronic lung disease‐PH algorithm.^13^ Features excluding use of inhaled treprostinil such as active cigarette smoking were also relevant to treatment decisions. With this consensus approach, *N* = 1 patient was classified as having left‐heart disease PH (WSPH Group 2 disease) based on RHC data consistent with post‐capillary disease and *N* = 1 as having combined pre‐ and postcapillary PH; diuretic therapy was prescribed for these patients (*N* = 2). We identified *N* = 3 additional patients as having PAH despite underlying lung disease and thereby deemed appropriate for alternative pulmonary vasodilator treatments; *N* = 1 patient required admission for initiation of parental prostacyclin therapy due to WHO Class IV symptoms, *N* = 1 was identified as having portopulmonary PH and nonspecific interstitial pneumonia and *N* = 1 active smoker was identified as having COPD and scleroderma *sine* (Figure 1). Of the three patients started on therapy, one patient with IPF was admitted to an outside hospital with an acute coronary syndrome within 3 weeks of therapeutic initiation (while on four puffs Q.I.D.) and expired. At initial follow‐up, a second patient (with COPD‐PH) titrated to six puffs Q.I.D., experienced a + 73.5 m improvement in 6MWD (baseline, 130 m; follow‐up, 203 m) and improvement from WHO‐FC IV to II. The third patient (COPD‐PH) experienced WHO‐FC improvement from III to II on six puffs Q.I.D. but efforts to advance treatment dose were limited by prohibitive cough.

## DISCUSSION

The recent approval of inhaled treprostinil therapy for ILD‐PH marks a long‐awaited change in the treatment landscape for lung disease‐PH; however, applying clinical trial results to “real‐world” populations requires further study and guidance. Here, in a Veteran population enriched with lung disease‐PH, we demonstrate that a protocolized approach to patient assessment for inhaled treprostinil candidacy is likely to refine appropriateness of WSPH Group determinations and associated prescribing decisions. Half the patients referred for presumed lung disease‐PH were re‐classified as having WSPH Group 1 or Group 2 PH, requiring initiation of PAH or diuretic therapies, respectively. These findings underscore the critical importance of carefully performed RHC,^14^ an underutilized diagnostic tool,^15^, ^16^ in evaluating patients referred for consideration of PH therapy. In particular, the availability of inhaled treprostinil for lung disease‐PH highlights an opportunity for guidelines to expand current recommendations for invasive hemodynamic assessment in patients where isolated WSPH Group 3 disease is presumed.

These findings further emphasize the established discordance between randomized controlled trial and nontrial cohorts of lung disease‐PH patients in which nontrial cohorts are often older with higher prevalence of comorbid disease and would therefore have been ineligible for clinical trial enrollment.^17^, ^18^, ^19^ While the description of an older and predominately male Veteran population differs from the patients enrolled in INCREASE, reporting on this clinically vulnerable population with elevated morbidity risk^20^, ^21^ is critical, as previously highlighted in idiopathic PAH.^22^ Furthermore, a protocolized approach to the assessment of compassionate use prescribing in select COPD‐PH patients supports prescribers tasked with treatment decisions in this population and motivates ongoing prospective study of inhaled treprostinil in patients with obstructive lung disease. This may in turn, limit inappropriate off‐label prescribing as is common with PAH‐approved therapies in WSPH Group 3 PH.^23^, ^24^

Benefits of protocolized administration of PH‐specific therapy include generalizability across different PH centers as a key step toward ensuring accurate diagnosis, conscientious prescribing of costly therapies, and quality control of appropriate and effective use. Our standardized approach to inhaled treprostinil prescribing may function as an important clinical (and research) asset by which to profile real‐world efficacy of this proven, albeit expensive and patient‐intensive, therapy for lung disease‐PH patients. In fact, the crude cost of inhaled treprostinil prescriptions saved by deferring therapy for the 7 patients deemed more appropriate to receive alternative PAH or diuretic pharmacotherapies was estimated to be $1,149,750 per year in the VA system. To this end, we invite communication with providers and institutions interested in collaborative efforts to study real‐world use of inhaled treprostinil (please contact shelsey.johnson@bmc.org or bmaron@bwh.harvard.edu which may support future efforts to expand the range and detail of information collected by additional iterations of this quality control initiative.

## AUTHOR CONTRIBUTIONS

*Study concept and design*: All authors. *Acquisition of data*: Shelsey W. Johnson. *Analysis and interpretation of data*: All authors. *Drafting of the manuscript*: Shelsey W. Johnson and Bradley A. Maron. *Critical revision of the manuscript for important intellectual content*: All authors.

## CONFLICTS OF INTEREST

Bradley A. Maron: Actelion Pharmaceuticals (Outside the scope of the current work), Deerfield Company (Outside the scope of the current work), Tenax Therapeutics (Outside the scope of the current work). The other authors declare no conflicts of interest.

## ETHICS STATEMENT

The Boston VA IRB committee deemed this study to be exempt from IRB review. Additionally, the Boston VA privacy committee reviewed this manuscript and approved submission based on the reporting of deidentified patient data. Shelsey W. Johnson had full access to all the data in the study and takes responsibility for the integrity of the data and the accuracy of the data analysis.
